# Universal and uniquely human factors in spontaneous number perception

**DOI:** 10.1038/ncomms13968

**Published:** 2017-01-16

**Authors:** Stephen Ferrigno, Julian Jara-Ettinger, Steven T. Piantadosi, Jessica F. Cantlon

**Affiliations:** 1 Department of Brain and Cognitive Sciences, 358 Meliora Hall, University of Rochester, Rochester, New York 14627, USA; 2 Department of Brain and Cognitive Sciences, 77 Massachusetts Ave., MIT, Cambridge, Massachusetts 02139, USA

## Abstract

A capacity for nonverbal numerical estimation is widespread among humans and animals. However, it is currently unclear whether numerical percepts are spontaneously extracted from the environment and whether nonverbal perception is influenced by human exposure to formal mathematics. We tested US adults and children, non-human primates, and numerate and innumerate Tsimane' adults on a quantity task in which they could choose to categorize sets of dots on the basis of number alone, surface area alone or a combination of the two. Despite differences in age, species and education, subjects are universally biased to base their judgments on number as opposed to the alternatives. Numerical biases are uniquely enhanced in humans compared to non-human primates, and correlated with degree of mathematics experience in both the US and Tsimane' groups. We conclude that humans universally and spontaneously extract numerical information, and that human nonverbal numerical perception is enhanced by symbolic numeracy.

## Abstract (web summary)

Perception of numerical quantities has been demonstrated in humans and animals; however, whether this is a natural ability is not clear. Here the authors show that human children and adults as well as monkeys spontaneously use number over surface area to categorize dot stimuli and this preference is enhanced with numerical literacy.

Although humans are the only species capable of symbolic, verbal counting, substantial research indicates that other animals can nonverbally perceive numerical quantity across a range of naturalistic and experimental tasks^1^,^2^,^3^. For example, one commonly used nonverbal numerical task is a comparison task in which an animal must choose the numerically larger of two visual arrays of dots that differ in number but not in shape, colour, size or other perceptual dimensions^4^. If the animal chooses correctly, it receives a food reward. Several studies have shown success on such tasks by a variety of animals including species of primates, rodents and birds^4^,^5^,^6^,^7^. In all of those studies, the animals' sensitivity to differences in numerical value is relatively crude, when compared to the precision and accuracy of human verbal counting. Monkeys, for example, can succeed at choosing the larger set from comparisons of 4 versus 8, 6 versus 12 and 10 versus 20 (a 2:1 ratio) but they typically fail at choosing the larger from 6 versus 8, 16 versus 20 and 20 versus 26 (a 3:4 ratio) (refs 4, 5). The best predictor of animals' numerical discrimination accuracy is the ratio between the quantities they are tasked with comparing. This psychophysical pattern is known as Weber's law and it is a hallmark of numerical estimation^3^. The ability to estimate numerical values may be an important evolutionary precursor to human counting since it is one of the most fundamental representations of quantity in the animal kingdom.

The ability to estimate the numerically larger or smaller set from among options is also present in human infants, children and adults. Nonverbal numerical estimation is present in infancy^8^,^9^,^10^,^11^ and as early as 2 days postnatal^9^. In studies that use looking time as the dependent measures, babies have been shown to look longer at sets that suddenly increase or decrease in numerical value, provided that the numerical change exceeds a 2:1 ratio. Numerical estimation abilities persist into childhood and adulthood, regardless of whether or not an individual learns verbal counting or is able to use it in an experimental task^12^,^13^. Adults living in remote cultures where verbal counting is not routine also possess a capacity for numerical estimation that is comparable to adults in industrialized cultures^13^,^14^. Thus, nonverbal numerical estimation is a fundamental cognitive ability that spans human cultures and stages of development.

Numerical estimation facilitates humans' and animals' abilities to make foraging and social decisions^1^,^15^. However, despite the fact that numerical estimation appears to be a widespread ability, the world offers a much richer array of other quantitative dimensions that animals could access. Dimensions such as surface area, duration and density provide valuable quantitative information about which option, given a set of choices, has more. In nature, these dimensions are often correlated with number. For example, a set of six berries has a greater number of items and often takes up more space (that is, has greater cumulative surface area and volume) than a set of three berries. But, the relation is not perfect: a set of six large berries may take up more space than 12 small berries. Several studies have shown that human children, animals and even human adults are sensitive to multiple quantitative dimensions when they are quantifying sets of objects^4^,^16^,^17^,^18^,^19^.

The issue of whether number is a naturally dominant dimension in human nonverbal perception is currently unclear^19^,^20^,^21^. Overshadowing of one dimension by another is a common phenomenon that depends on a species' evolutionary history and environmental pressures^22^. Evolutionary constraints on primate perceptual systems may favour discrete object-based numerosity over surface dimensions like area and size. If so, number will be a fundamentally salient dimension for humans independently of age or cultural experience—and more generally for other primate species as well. Alternatively, perceptual systems may only learn to attend to number instead of other quantitative dimensions as a consequence of input from cultural numeracy.

To address this question, we tested the natural tendency to represent number using the same task across non-human primates, human children, US adults and adults from a predominantly low-numeracy cultural group in Bolivia, the Tsimane'^23^. This is the first direct comparison of number perception using a common task across such a diverse group of population samples. We tested the relative salience of number versus cumulative surface area using a spontaneous categorization task similar to previous research in child development and psychophysics (Fig. 1)^24^,^25^,^26^. Subjects first learned by rote association to categorize dot arrays into two categories (small quantity right side and large quantity left side or vice versa, Fig. 2a). Importantly, the number and cumulative area of the dot arrays were completely correlated during the training so subjects could use either or both dimensions as the basis for their category choices. Individual dot size was controlled by equating individual dot sizes between all training stimuli. In order to keep the task the same across groups, no verbal description of the categories was provided to any of the subjects. Instead, subjects learned the categories from nonverbal demonstration by the experimenter and trial-and-error feedback (see Methods for complete details).

Once subjects were trained to accurately categorize the training stimuli, a small percentage of ‘probe' stimuli were added in with the presentation of ‘standard' training stimuli. On these probe trials, number and cumulative area were systematically uncorrelated so that responses to these stimuli would show which dimension subjects spontaneously used (Fig. 2). On probe trials, all responses were treated as correct and positively reinforced. This procedure allowed us to precisely measure the contribution of each dimension to subjects' underlying categorization of the quantities. If number was the primary basis of subjects' categorization judgments during training, then as number of dots increases, the percentage of trials where subjects select the ‘more' category should also increase, regardless of the cumulative area of the dots. In contrast if surface area was the primary basis of categorization then subjects' choice of the ‘more' category will increase with the cumulative surface area values of the sets. If both dimensions were used relatively equally, subjects' category choices would be modulated to a similar degree by the number and cumulative surface area values. We found that all groups had a bias to use the numerical dimension of the stimuli regardless of age, culture or species. The number bias was greatly enhanced in humans compared to non-human primates and correlated with age and math education in humans.

## Results

### All groups performed the training task

Overall, monkeys, US children, Tsimane' adults and US adults all performed above chance on the ‘standard' training trials where number and cumulative surface area were correlated (one-sample Wilcoxon tests; US adults: mean=92%, W=252, *P*<0.001; Tsimane' adults: mean=90%, W=1,429, *P*<0.001; US children: mean=76%, W=947, *P*<0.001; Monkeys by session: mean=78%, W=561, *P*<0.001). Subjects who did not reach above chance levels on the training trials according to a binomial test (∼60% correct) were excluded from further analyses (5% of US adults, 6% of Tsimane' adults and 26% of US children).

### Fundamental bias to represent numerical information

In order to determine the relative contributions of number and cumulative area to subjects' quantity judgments, we conducted a mixed effects logistic regression using number and cumulative area as predictor variables of category choice, and a random effects term of subject for each group (equation (1), see also Methods for details on data analysis)^27^. This random effects approach is powerful because it tests for the simultaneous influence of each predictor while protecting against finding effects at the group level that are not represented at the individual subject level. This approach also produces more accurate and conservative error terms than fixed effects regression models. In the analysis, effects of number and cumulative area are orthogonal, such that for any number effect, area is controlled for and any area effect, number is controlled.


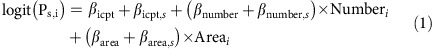


**Figure 3:**
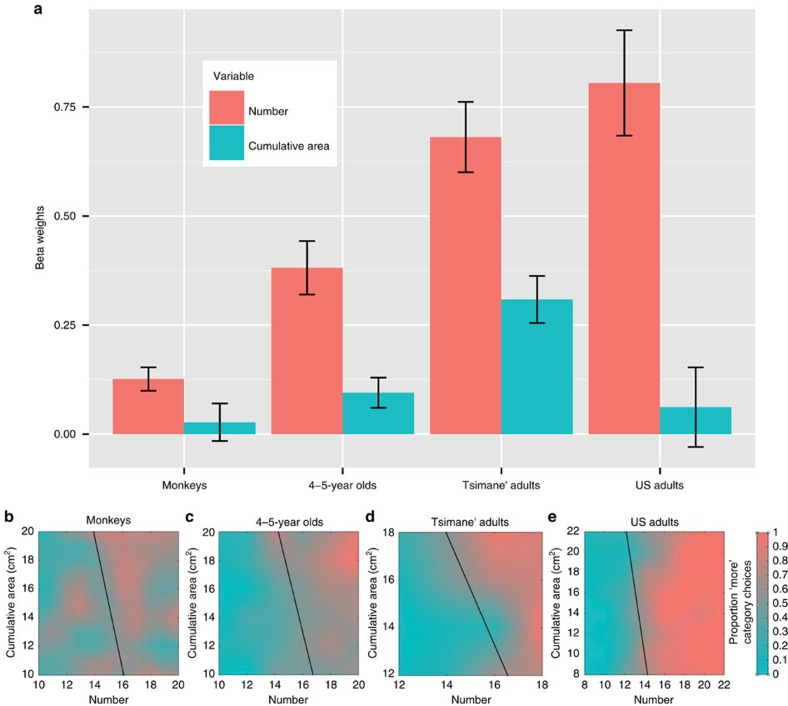
Relative contributions of number and cumulative area to performance. (**a**) Beta weights from a mixed effects logistic regression predicting category choice using number and cumulative area as predictor variables and including subject as a random effect (US adults: *n*=21; Tsimane' adults: *n*=51, 4-5-year-old US children: *n*=27 and monkeys (*n*=2). Error bars represent the standard error of the mean. Heat maps and a linear classifier of categorization performance for (**b**) monkeys, (**c**) 4-5 year-old children, (**d**) Tsimane' adults and (**e**) US adults show the category boundaries of each group.

First, we found that number significantly predicted category choice in all groups. Cumulative area had a positive effect on performance in all groups but only reached significance in the 4-5-year-old children and Tsimane' adults (Fig. 3a and Table 1). The relative contribution of each dimension to subjects' performance is measured by its beta weight (Fig. 3a). Each beta weight quantifies the amount by which changes in each stimulus dimension influence the behavioural categorization choice. A direct comparison of the number and cumulative area beta weights revealed that number had a significantly greater effect than cumulative surface area on subjects' choices in all groups (*z*-test: US adults: *z*=4.91, *P*<0.001, *n*=2,436; Tsimane' adults: *z*=3.84, *P*<0.001, *n*=714; US 4-5-year-olds: *z*=4.07, *P*<0.001, *n*=864; Monkeys: *z*=1.96, *P*=0.05, *n*=380). A regression with logarithmically transformed predictors yielded qualitatively similar results (Supplementary Fig. 1).

The subjects' category representations can be visualized by calculating a decision boundary from the beta weights as shown in Fig. 3b-e; see equation (2). Lines closer to vertical (90°) reflect decisions based primarily on number, and lines closer to horizontal (0°) reflect decisions based primarily on cumulative area. All groups had slopes that were more vertical than horizontal (>45°), showing that for all groups performance was more affected by the numerical dimension than by cumulative surface area. When we compared slopes between groups we found that US adults had the most vertical slope (−12.99, 85°), US children and monkeys had similar slopes (US children=−4.01, 76°; Monkeys=−4.63, 78°), and Tsimane' adults had the least vertical slope of all the groups (−2.21, 66°). This is because although Tsimane' adults were influenced by number almost as much as the US adults, they showed greater influence of cumulative area on their decisions. All groups showed a weak trend for representing cumulative area in addition to number. When all groups were combined we saw a small, but significant effect of cumulative area on category judgments (*β*_cumulative area_=0.06, *P*<0.001, *n*=4,394). For this combined analysis, we used a random effect term of group. This effect was about one fifth as strong as the effect of number on categorization performance (*β*_number_=0.33, *P*<0.001, *n*=4,394). These results show that all groups represented the numerical dimension.


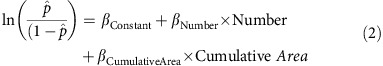


### Species and maturity affect the number bias

**Figure 4:**
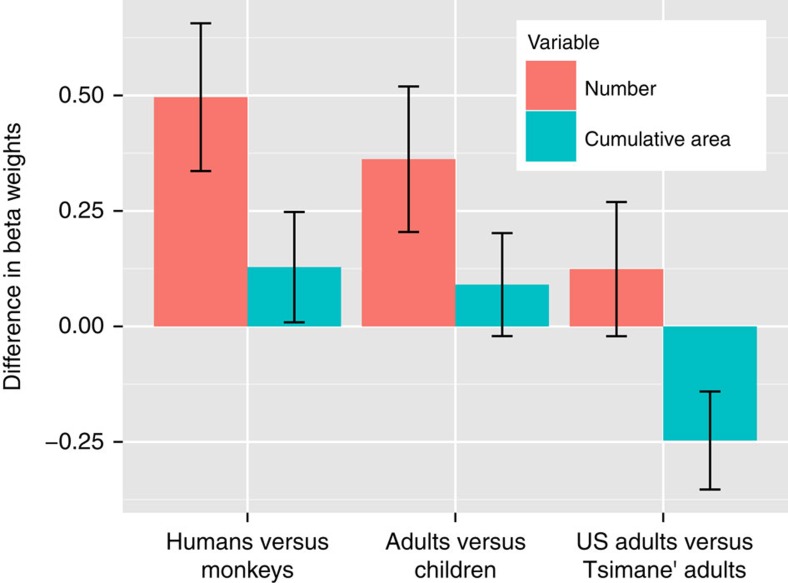
Group differences in number and cumulative area categorization biases. The greatest differences in number perception are driven by species (humans versus monkeys) and age (adults versus children), not culture (US adults versus Tsimane' adults). Comparisons were calculated by averaging coefficients from separate regressions and then comparing across groups (US adults: *n*=21; Tsimane' adults: *n*=51, 4-5-year-old US children: *n*=27; Monkeys *n*=2). Error bars represent the standard error of the mean and were approximated using the method of Clogg *et al*.^28^

We quantified the degree to which the number bias was influenced by species, age and cultural numeracy through group comparisons. To keep subject effects fit within each population, we averaged the coefficients (from Fig. 3) from each separate regression across several key contrasting groups: humans and non-humans, adult humans and human children, and low and high numerate adults. The errors on each contrast were approximated using the method of Clogg *et al*.^28^ and statistical reliability was assessed with *z*-tests (equation (5), Fig. 4 and Supplementary Table 1). We found the largest group difference was between humans and non-humans. Overall, humans had stronger number-based category weights than non-humans, but there was no significant difference in area-based categorization. Among humans, we found significant effects of maturity. Adults had stronger number-based categorization compared to human children, but there was no significant difference in area-based categorization. We found no significant difference in number-based categorization between U.S. adults and Tsimane' adults. There was a small but significant difference in area categorization, with Tsimane' adults factoring area more strongly than US adults. Thus, the greatest differences in nonverbal number representations between groups were related to species and maturity, not culture.

### Math education enhances the number bias in humans

**Figure 5:**
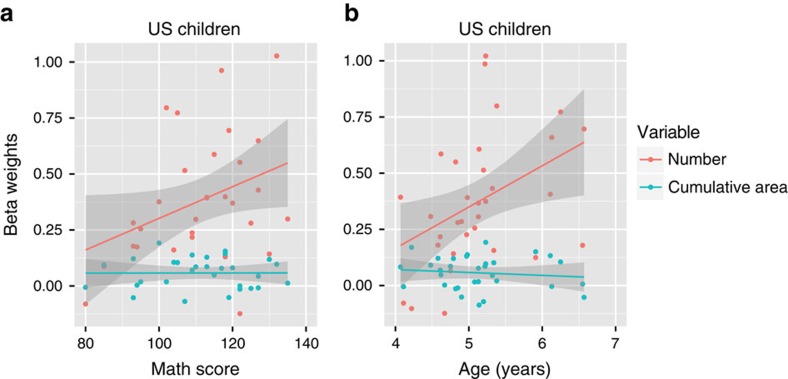
Effects of age and mathematics education on number representations in US children. Age and mathematics ability are related to perception of number but not cumulative surface area. The figure plots the beta weights from a mixed model logistic regression showing (**a**) the effect of age and (**b**) the effect of math ability (standardized score) on number and area categorization in US children (*n*=32). Shaded regions represent 95% confidence intervals.

In order to measure the effects of mathematics enculturation within children, we conducted a mixed effects logistic regression, which included standardized math ability (measured by the TEMA- Test of Early Mathematics Ability), age, number and area weights as predictors for the categorization performance during probe trials and a random effects term of subject (see equation (6)). We also included interaction factors of standardized math ability and age on both the number and area coefficients. This allowed us to measure the specific effects of math ability and age on US children's number and area representations. If age or math ability enhances the natural representation of number or area, we should see that increases in age and/or math ability lead to increases in the beta weight for that dimension, an effect that shows up as an interaction in the regression. We found that formal mathematics ability had a significant positive interaction with number representation (Fig. 5a, Supplementary Table 2), even when controlling for age. In contrast, math ability had no interaction effect with surface area representation. This suggests that math ability increases subjects' natural tendency to perceive number as a distinct dimension. Additionally, we found an independent effect of age, such that as age increased, so did children's bias to use number as the basis of their category judgments, even when standardized mathematics differences were controlled (Fig. 5b and Supplementary Table 2). This age effect is most likely due to a relation between number perception and age-dependent math millstones such as learning to count, order and add numbers. Thus, number perception is robust across age groups, but the representation is substantially enhanced by formal mathematics learning in children.

**Figure 6:**
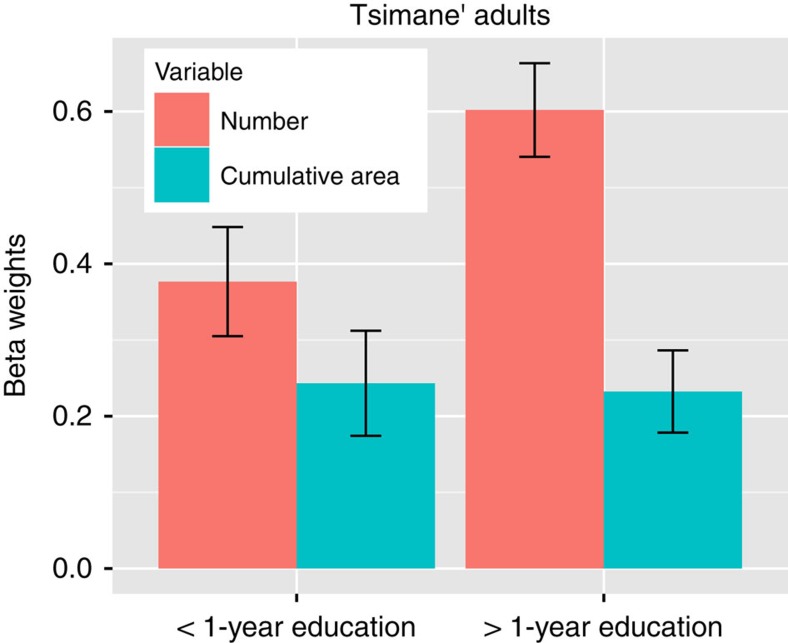
Effects of education on number representations in Tsimane' adults. Tsimane' adults show an effect of education on the perception of number but not cumulative surface area. The beta weights were calculated from a mixed model logistic regression showing the effect of education on number and area weights in Tsimane' adults (*n*=51). Error bars represent the s.e. of the mean.

An analysis of the effects of education on the number and area category judgments in the Tsimane' adults revealed similar results (see equation (7)). We found an interaction between years of education and number-biased categorization. As education increased, subjects were more likely to classify the stimuli based on number (Supplementary Table 3). This linear correlation was trending but not significant, likely due to imprecision in Tsimane' estimations of their years of education. In order to confirm this relation, we conducted an additional analysis grouping Tsimane' adults by <1 year or >1 year of education. We found a significant interaction between education group (<1 year of education and >1 year of education) and number-based categorization (*β*_education group*number_ =0.32, *P*<0.05, *n*=714; Fig. 6). This analysis provides more conclusive evidence that education level was related to the nonverbal number bias in the Tsimane' adults. The results show that even in adults, formal mathematics education enhances humans' nonverbal numerical perception.

### Number bias is stronger than alternatives

Previous research has shown that other continuous dimensions can be used to represent quantities such as density, contour length, convex hull (the smallest contour around the dot array) brightness and individual element size^16^,^29^,^30^. Each of these strategies would produce a distinctive pattern of categorization in our task. If subjects used density then we should see a significant effect of density on performance. We defined density as number of dots per unit area in the convex hull of the dots. Density and number were entered as predictor variables of subjects' category choices (standardized; equation (8)). We also included a random effects term of group. We found no significant effect of density. Instead we found a significant effect of number over and above density (*β*_density_=0.03, *P*=0.77, *n*=4,785; *β*_number_=1.22, *P*<0.001, *n*=4,785). We also found no decrease in number weights when density was controlled in a separate experiment, Experiment 2 (Supplementary Note 1 and Supplementary Methods).

Next, we tested the effects of contour length, convex hull and individual element size in Experiment 1 (equations (9)–(11), 10, 11). We used standardized variables and a random effects term of group for each analysis. We found no effect of convex hull (*β*_convex hull_=0.01, *P*=0.89, *n*=4,785; *β*_number_=1.25, *P*<0.001, *n*=4,785). There was a small effect of contour length, but the independent effect of number was almost ten times greater than the effect of contour length (*β*_contour length_=0.15, *P*=0.03, *n*=4,785; *β*_number=_1.13, *P*<0.001, *n*=4,785). When individual dot size and number are used as predictors for category choice, we see a small effect of dot size (*β*_individual dot size_=0.30, *P*<0.001, *n*=4,785; *β*_number_=1.49, *P*<0.001, *n*=4,785), but this is likely due to covariance between dot size and cumulative area. When cumulative area is added to the regression, the effect of dot size is reduced but the number effect remains strong (*β*_individual dot size_=−0.26, *P*=0.06, *n*=4,785; *β*_number_=1.13, *P*<0.001, *n*=4,785; *β*_area_=0.42, *P*<0.001, *n*=4,785). Additionally, if subjects used individual dot size, then the slopes of the linear classifiers (Fig. 3b–e) would be positive. Instead all groups showed negative slopes. Brightness was correlated with cumulative area, such that the cumulative area term would account for any use of brightness. Thus brightness could not explain the effect of number. The results conclusively show that subjects' quantitative choices were rooted in numerical perception.

Some researchers have suggested that a combination of non-numeric dimensions is used to quantify sets of objects, instead of representing number directly^31^. To test this we implemented a regression analysis, which included the combination of variables suggested (convex hull, density, cumulative surface area and average diameter) and also included number (equation (12)) on our results from Experiment 1 (ref. 31). Again group was entered as a random effects term. If number is represented via these alternative dimensions then we should find *no* significant effect of number when this collection of variables is included in the model. This is not what we found. Instead, number was a significant predictor over and above all other measures (*β*_number_=1.05, *P*<0.001, *n*=4,785). This persistent effect of number even when other variables are controlled shows that the effect of number cannot be accounted for by alternative variables. Additionally, the effect of number was significantly greater than any of the variables tested (*z*-tests: Number versus Convex Hull: *z*=7.39, *P* <0.001, *n*=4,785; Number versus Density: *z*=2.79, *P*<0.01, *n*=4,785; Number versus Area: *z*=2.15, *P*<0.05, *n*=4,785; Number versus Dot Diameter: *z*=5.51, *P*<0.001, *n*=4,785). The results suggest that numerical information is perceived directly, rather than indirectly through the representation of other quantitative dimensions^32^,^33^.

## Discussion

The current study tested the hypothesis that humans spontaneously perceive the numerical dimension of a stimulus as a consequence of both evolutionarily primitive constraints on perception and cultural input. Our results show clear effects of an evolutionary influence on number perception in the widespread number biases observed across different primate species, age groups and human cultures. Our results also show an effect of cultural numeracy on nonverbal number perception in the correlation between education and number bias in US children and Tsimane' adults. Together these data provide the first direct assessment of the relative contributions of evolution and culture to human nonverbal number perception.

This evidence of universal number perception supports the theory that number is a naturally salient dimension that requires no specific cultural input^4^,^11^,^34^,^35^,^36^,^37^. Previous research has shown that people from cultures with limited number knowledge or even number words can use number to represent sets of objects when other continuous dimensions are controlled^13^,^14^. A recent neuroscience study found number-specific neural responses in the parietal cortex of untrained monkeys—further evidence of spontaneous number representation in primates^38^,^39^. Our results expand on prior reports by showing that number perception emerges spontaneously, even when alternative quantitative dimensions are available as the basis for judgment, in humans of various ages and cultures and even in non-human primates. These results indicate that evolutionarily primitive constraints on primate perception influence spontaneous number perception in humans, regardless of cultural experience.

Although we suggest that the robust number bias we observed across groups originates from fundamental perceptual representations, a non-exclusive alternative explanation is that number and area weights in categorization judgments were based on a shift of attention or task-set from one dimension to the other. Under this explanation, subjects represented number-based and cumulative area-based categorization rules equally well and shifted attention or explicitly selected one task-rule over another. If this were the case then we would expect that as number weights increase, area weights would decrease—however, this trade-off pattern was not observed. There was no significant decrease in area weights as number weights increased, and no correlation between area weights and education or age (Figs 5 and 6 and Supplementary Tables 2-3). Instead we saw that the number and area coefficients were independent. There was no correlation between number and cumulative area beta weights across subjects (linear correlation: Tsimane' adults: *R*^2^=0.001, *P*=0.84, *n*=51; US children: *R*^2^=0.061, *P*=0.183, *n*=32). The effects are not likely due to fluid shifting of attention or rules from one dimension to the other. Thus, although there is still room to investigate interactions between sensitivity and bias in number perception across groups, our data show that the number bias is attributable to variability in the perception of number that is independent of variability in surface area perception. Moreover, the commonalities in performance between monkeys and humans, and across humans from different ages and cultures indicate that this cognitive phenomenon is robust and unlikely to be caused by only temporary or superficial factors.

Secondly, our results isolate the effects of species, age and cultural learning on human nonverbal numerical representation. The largest factors influencing human nonverbal numerical perception were species and age, suggesting that numerical perception is enhanced in humans due to unique aspects of their development. Cultural differences between the US adults and Tsimane' adults that derive from immersion in a numerate or non-numerate society were only associated with small differences in spontaneous quantity perception. However, within US and Tsimane' groups, formal mathematics education had a transformative effect on the spontaneous perception and use of number. The perceptual salience of number increased with exposure to formal numerical concepts for both US children and Tsimane' adults. This effect is likely directional from education to nonverbal number perception particularly in the Tsimane' for whom the measure of education is based on circumstantial exposure to formal mathematics rather than mathematics ability. Since a nonverbal number bias is unlikely to provide Tsimane' individuals with greater access to mathematics education, the most likely explanation of the relation is that exposure to cultural mathematics enhanced the nonverbal perception of number. Previous research has suggested that humans' primitive nonverbal number capacities influence their formal, symbolic mathematics abilities^40^,^41^,^42^. Our data provide novel casual evidence that nonverbal perception of number is enhanced by the construction of formal knowledge systems in humans. Together with previous findings, our results suggest that primitive and formal numerical concepts mutually enhance each other in human cognition. Interactions between number representations are likely bi-directional, such that early nonverbal number capacities influence verbal mathematics concepts^40^,^41^,^42^ and exposure to mathematics enhances nonverbal number capacities as shown in our results^43^.

Finally our data show that number has a strong influence on subjects' perceptual categorization even when accounting for variability in other dimensions—suggesting that number is perceived directly instead of indirectly as suggested by some previous research^16^,^29^,^30^,^31^. These results raise the question of why number is such a fundamental and universal percept. One possible explanation is that primate perception is object-oriented (for example, more primate cortex dedicated to objects than surfaces^44^,^45^) and that number is a quantitative dimension that operates over discrete objects. These fundamental constraints on information processing might make numerical quantities more salient than other continuous and surface-based quantitative dimensions in primate cognition. In fact, previous research with human infants has shown that infants find numerical quantities easier to extract from a set of objects than surface quantities^46^,^47^,^48^. Infants can detect quantitative changes in number and surface area but they require a proportionally greater change in surface area compared to number to detect those changes. If number and area were equally weighted in infant perception then number and surface area would be equally easy to discriminate on a ratio scale. Our finding that even monkeys are biased to represent number over surface area suggests that previous findings from human infants could be part of a more general phenomenon—that evolutionary constraints on primate perception are responsible for the more robust perception of number compared to surface area in humans. The primacy of numerical perception across primate species, age and culture may be specific to the visual system, as reported here, or it could extend to other modalities^49^,^50^,^51^,^52^. A fundamental bias to segregate numerical information from other quantitative dimensions and *use* it preferentially was likely an important catalyst for the emergence of an abstract, discrete counting system in human cultural evolution.

## Methods

### Participants

Sample sizes for human subjects were designed to match the amount of data collected between groups based on the number of task trials that subjects from each group could reasonably complete. US adult participants were 22 adults (mean age=22.6, standard deviation=6.5, 7 males). Participants were undergraduate students recruited from the University of Rochester River Campus. All guidelines and requirements of the University of Rochester's Research Subjects Review Board were followed for participant recruitment and experimental procedures. Informed consent was obtained from all subjects. One US adult was excluded from analysis because performance on the training trials did not reach significance at the *P*=0.05 level (60% correct), indicating that they did not understand the general task.

US child participants were 47 children (mean age: 5.1 years, age range: 4.1–6.6 years, 26 male). Participants were recruited from Rochester and surrounding areas to the Kid Neuro Lab at the University of Rochester. All guidelines and requirements of the University of Rochester's Research Subjects Review Board were followed for participant recruitment and experimental procedures. Informed consent was obtained from all subjects' parents or guardians. U.S. children completed the TEMA-3 test of early mathematics ability^53^. All children showed scores within a normal range for typically developing children. Twelve US children were excluded from analysis because performance on the training trials did not reach significance at the *P*=0.05 level (60% correct), indicating that they did not understand the general task. Three additional children were excluded because they fussed out of the experiment.

Tsimane' adult participants were 54 adults (mean age=32.4 years, standard deviation=15 years, 10 males). Participants were recruited from five Tsimane' communities near San Borja, Bolivia. All guidelines and requirements of the University of Rochester's Research Subjects Review Board were followed for participant recruitment and experimental procedures. Informed consent was obtained from all subjects. Interpreters were provided by the Centro Boliviano de Investigación y de Desarrollo Socio Integral. Subjects had a range between 0 and 10 years of formal education taught at the local village (mean: 2.6 years). This measure of education was used to split subjects into low and high education groups because subjects only approximated their educational history. Three Tsimane' adults were excluded from analyses because performance on the training trials did not reach 60% correct, indicating that they did not understand the general task.

Nonhuman primate subjects were three adult female rhesus macaques (age=6 years), who were socially housed. Animals were kept on a water-restricted diet approved by the University of Rochester Committee on Animal Resources and veterinary staff. All animal care procedures were in accordance with an IACUC protocol. All monkeys had prior experience with matching tasks using photographs and geometric shapes. One monkey had experience matching visual arrays in which number and area were correlated as in the current training protocol. One monkey was excluded from analyses because she used an idiosyncratic strategy. The role of the probe trials was to determine which stimulus features were being used to form the categories during the standard trials. This subject used a novel dimension (dot size) only on probe trials, which could not have been used during the standard trials (because dot size was equated across all stimuli) Thus, we were unable to determine how this animal's categories were initially formed or represented. This was established by a logistic regression in which number of dots, cumulative area of dots and individual dots size were entered as predictors of category choice (*β*_number_=0.41, *P*<0.01; *β*_cumulative area_=−0.18, *P*=0.18; *β*_dot size=_7.96, *P*<0.001, *n*=232). This strategy could not have been used during training because individual dot size was held constant and homogeneous for all training stimuli. Moreover, the subject changed strategies between standard and probe trials. During standard trials, the subjects had a significant effect of number (*β*_number_=0.27, *P*<0.001, *n*=2,136), whereas during the probe trials the direction of this effect was reversed (*β*_number_=−0.12, *P*<0.001, *n*=232). Since dot size was inversely correlated with number only on test stimuli, the positive effect of dot size only on test trials could indicate that this monkey used ‘number' throughout training and testing but mistakenly switched the categories at test. In either case, the animal adopted an anomalous, inconsistent task strategy. Importantly, even when we include this subject in the random effects analysis, we find that ‘number' has a greater effect on performance than ‘cumulative surface area' and all other dimensions (*β*_number=_1.23, *P*<0.001; *β*_cumulative area_=0.47, *P*<0.001; *β*_convex hull_=−0.13, *P*<0.01; *β*_density_=−0.60, *P*<0.001; *β*_dot diameter_=0.15, *P*=0.51, *n*=4,786). The results still support our main conclusion. Since this animal anomalously used qualitatively different solutions on probe stimuli versus training stimuli, we present her results here.

### Materials

Stimuli were presented on an Elo (ET1529L) touchscreen monitor for US children, adults and monkeys using Xojo (REALbasic). Stimuli were presented to Tsimane' adults on laminated 4″ × 4″ prints. Stimuli were created using Psychtoolbox (MATLAB).

The training and standard stimuli consisted of five picture types for each category. For category A, the training stimuli had 8, 9, 10, 11 or 12 dots. All of the dots during the training stimuli had an individual dot area of 1 cm^2^; thus these arrays had a cumulative surface area of 8, 9, 10, 11 or 12 cm^2^. For category B, the stimuli had 18, 19, 20, 21 or 22 dots. These pictures had a cumulative surface area of 18, 19, 20, 21 or 22 cm^2^. There was an equal number of category A and B stimuli in the training trials. Training and standard stimuli were sampled from a normal distribution of the category such that each category had approximately 15% at 8 or 18, 21% at 9 or 19, 27% at 10 or 20, 21% at 11 or 21 and 15% at 12 or 22. The placement of the dots for each stimuli was randomized, such that no subject saw the same placements of dots more than once.

During probe trials, the number of dots and cumulative area of dots were systematically uncorrelated in a full cross of the two dimensions. The stimulus space was uniformly sampled for all subjects such that the average number and cumulative area was 15 dots and cm^2^ (Range: US adults: 8-22, US children: 10-20, Tsimane' adults: 12-18, monkeys: 8-22). Additionally, this sampling was orthogonal such that the number of dots presented did not change the likelihood of the area of dots presented and the area of dots presented did not change the likelihood of the number of dots presented. For each number of dots, all cumulative area values were tested, and for each cumulative area value, all numbers of dots were tested (see Fig. 6a). Number and cumulative area values ranged from 8 to 22 (8, 10, 12, 14, 16, 18, 20, 22 dots and cm^2^ cumulative area).

### Procedure

The procedure was selected such that it was highly similar between groups, yet tailored to be appropriate for each of the populations tested. All US children, US adults and monkeys were presented with the task using touch screen monitors. Subjects initiated a trial by touching a start stimulus, a small white box in the centre of the screen. Once a trial was initiated, an array of dots was then presented in the centre of the screen. Subjects were required to press this stimuli causing it to be replaced by two symbols on the left and right side of the screen. These symbols were arbitrary category symbols (star and diamond) each representing either the ‘Less' category or the ‘More' category. Both symbols had the same total cumulative area. The left and right placement of the category symbols was randomized as well as which category each symbol represented between subjects. If subjects pressed the matching category symbol for a standard training stimulus they would receive positive auditory feedback (and food reward for monkeys). This was then followed by a 1 s inter-trial interval before the next trial could be initiated. If subjects pressed the non-matching category symbol on the standard training stimuli, they received negative auditory feedback and a black time out screen for 2 s followed by the inter-trial interval (see Fig. 6b). No differential feedback was given on probe trials.

Due to the lack of familiarity with computers all Tsimane' adults were presented with stimuli printed on 4″ by 4″ index cards. The two arbitrary category symbols (star and diamond) were placed in front of the subjects. Cards with dot arrays on them were then held up for the subjects to see. Subjects would then point to one of the category symbols. If the correct category was chosen, subjects would receive positive verbal feedback ‘correct' only on standard trials. If the incorrect category was chosen they would receive negative verbal feedback ‘incorrect' on standard trials. As for other groups, no feedback was given on probe trials. The stimuli, randomization procedure and instructions were the same as in US groups.

### Training phase

All subjects received training on the standard stimuli to measure their initial categorization abilities when number and area were correlated. All subjects had to reach the same 60% criterion on training trials before receiving probe trials. Subjects were trained on a ratio scale of number and area that was on approximately 2:1 (8-12 versus 18-22 items and cm^2^). In all training stimuli the number of dots and cumulative area of the dots was 100% correlated. The individual dot size was held constant and homogeneous for all training stimuli.

In order to keep the task the same across groups, human subjects were not given any verbal labels for the categories. Human subjects were shown one exemplar from each category and told ‘this [showing small quantity] goes here and that [showing large quantity] goes there'. The training criterion was determined based on performance (60% accuracy) rather than trial count. US adults and children completed 30 initial training trials. We determined from prior research that Tsimane' adults were only comfortable with short experiments of not more than 50 trials, including training and testing, and so their initial training session was limited to 18 trials. However, all human subjects from US and Tsimane' groups met the 60% accuracy criterion on the training stimuli.

Because verbal instructions could not be provided to the monkeys, a familiarization phase was given. Monkeys were first trained to categorize the exemplars of the training categories (10 and 20 dots and total cumulative area in cm^2^) before the full set of training stimuli was given (Monkey 1: Sessions to criterion=24, Trials=3,321). Training continued until subjects reached 70% accuracy on the full set of training stimuli (Sessions to criterion=3 ,Trials=450). Monkey 2 did not learn to correctly classify the training trials at the 8-12 versus 18-22 classifications and was trained and tested on a 4:1 ratio scale, 8-12 versus 42-48 (Monkey 2: Exemplar sessions to criterion=38, Trials=5,721; Full training session to criterion=9, Trials=1,350). Number and cumulative area were still 100% correlated; thus, this difference could not bias the animal towards number or area.

### Testing phase

After reaching 60% accuracy on the training phase, a portion of probe trials was randomly intermixed with standard stimuli (Percent Probes: US adults: 38%, US children: 38%, Tsimane' adults: 50%, monkeys: 10%). The proportion of probe trials was selected such that the task would be appropriate in length for each group. We also chose the percentage of probe trials to ensure that subjects maintained high levels of accuracy throughout testing. Tsimane' adults received a slightly higher portion of probe trials (50%) compared to US subjects (38%) in order to collect enough probe data to sample the stimulus space for each subject within the maximum session length feasible for Tsimane' testing. For monkeys, we stretched the testing trials across more sessions with a lower percentage of probe trials (10%) so that they would not extinguish their training strategy in the presence of non-differential reinforcement on probe trials (probe trials were rewarded no matter what response monkeys made). Non-human primates are sensitive to changes in reward rates. Thus we wanted to minimize the percentage of non-differentially reinforced probe trials per session to minimize differences in reward rate between training and testing sessions. Monkeys completed the testing phase over an average of 20 sessions consisting of 135 training trials and 15 probe trials per day. US adults, US children and Tsimane' adults completed the testing phase in one session which consisted of 186 standards and 116 probe trials for US adults, 51 standards and 32 probe trials for US children, and 14 standards and 14 probe trials for Tsimane' adults.

### Data analysis

Mixed effects logistic regressions were conducted to calculate how much each dimension was used to classify the probe trials^27^. This regression approach permits us to quantify the influence of multiple predictors on a binary behavioural outcome (response classification) while correctly handling the variability that arises from individual subject and group differences. This allows us to test for performance effects that are represented at the subject level and not just in the group average^54^. In all within-group analyses, a random effects term (Number + Cumulative Area|Subject) was used in order to capture differences in the effect of number and cumulative area for individuals. The resulting overall statistics we report reflect group effects that are similar to the average of within-subject regressions, but ‘pool' information across subjects and groups in a more efficient way, which helps to more accurately model error. The random effects term also helps to handle the different sample sizes. For ease of interpretation, all terms were first centred, but not standardized when variables were on the same scale (and had the same mean and s.d.). For the probe trials, number and cumulative area were orthogonal such that all values of cumulative area were tested for each number and all numbers were tested for each value of cumulative area. This allowed us to independently measure the contributions of each dimension, while controlling for the other. Throughout these analyses all terms were entered simultaneously in the model. This allowed us to measure the effects of one variable at the average of the other variable.

We used the output of the mixed effects regression described above to compare beta coefficients between groups, and examine the effects of species, maturity and culture on number representation. We averaged the coefficients from each separate group regression across several key contrasting variables: humans and non-humans, adult humans and human children, and low and high numerate adults. The errors on each contrast were approximated using the method of Clogg *et al*.^28^, and statistical reliability was assessed with *z*-tests. Differences in sample size between groups were accounted for by the random effects term and do not impact the effect size—thus coefficients are comparable between groups.

For the analyses of density, contour length, convex hull and element size, a random effects term of group (US adult, Tsimane' adult, US children, Monkeys) was used to provide beta weight estimates that correctly handle the variation between groups such that we could make generalizations about the effects that held across groups. All analyses were done using R statistics software and the LME package and the BOBYQA optimizer^55^,^56^.

We used mixed effects logistic regression equations of the general form:


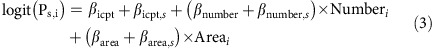


where *β*{number,*s*}, *β*{area,*s*}, *β*{icpt,*s*} are by-subject adjustments to the overall number effect (*β* {number}), area effect (*β*{area}) and intercept (*β*{icpt}), respectively. The outcome *P*{*s,i*} is the probability of responding in agreement with the Number category on the *s*th subject's *i*th trial. This form of mixed effect/hierarchical regression is similar to fitting a regression within each subject and then averaging the resulting coefficients. However, it handles the statistics in a more robust and correct way, and also allows greater flexibility in testing hypotheses^27^. Essentially, it provides the regression version of a paired (within-subjects) analysis. In the regression literature, this equation is often written as ‘Category ∼ Number + Area + (1 + Number + Area | Subject)' where the first part, ‘Number + Area' means that we are computing overall averages of coefficients and the second part ‘(1 + Number + Area | Subject)' means that we are adjusting these coefficients by (‘|') subject. The following regressions were used:


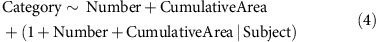



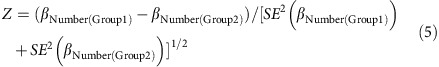



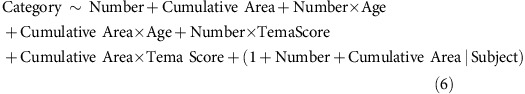



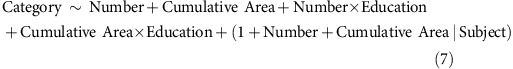



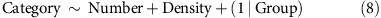



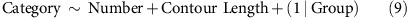



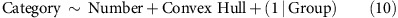



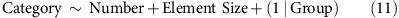



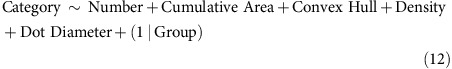


### Data availability

The data set used for this study is available from the corresponding author on request.

## Additional information

**How to cite this article:** Ferrigno, S. *et al*. Universal and uniquely human factors in spontaneous number perception. *Nat. Commun.* **8,** 13968 doi: 10.1038/ncomms13968 (2017).

**Publisher's note:** Springer Nature remains neutral with regard to jurisdictional claims in published maps and institutional affiliations.

## Supplementary Material

Supplementary Information Supplementary Figures, Supplementary Tables, Supplementary Notes, Supplementary Methods and Supplementary References

## Figures and Tables

**Figure 1:**
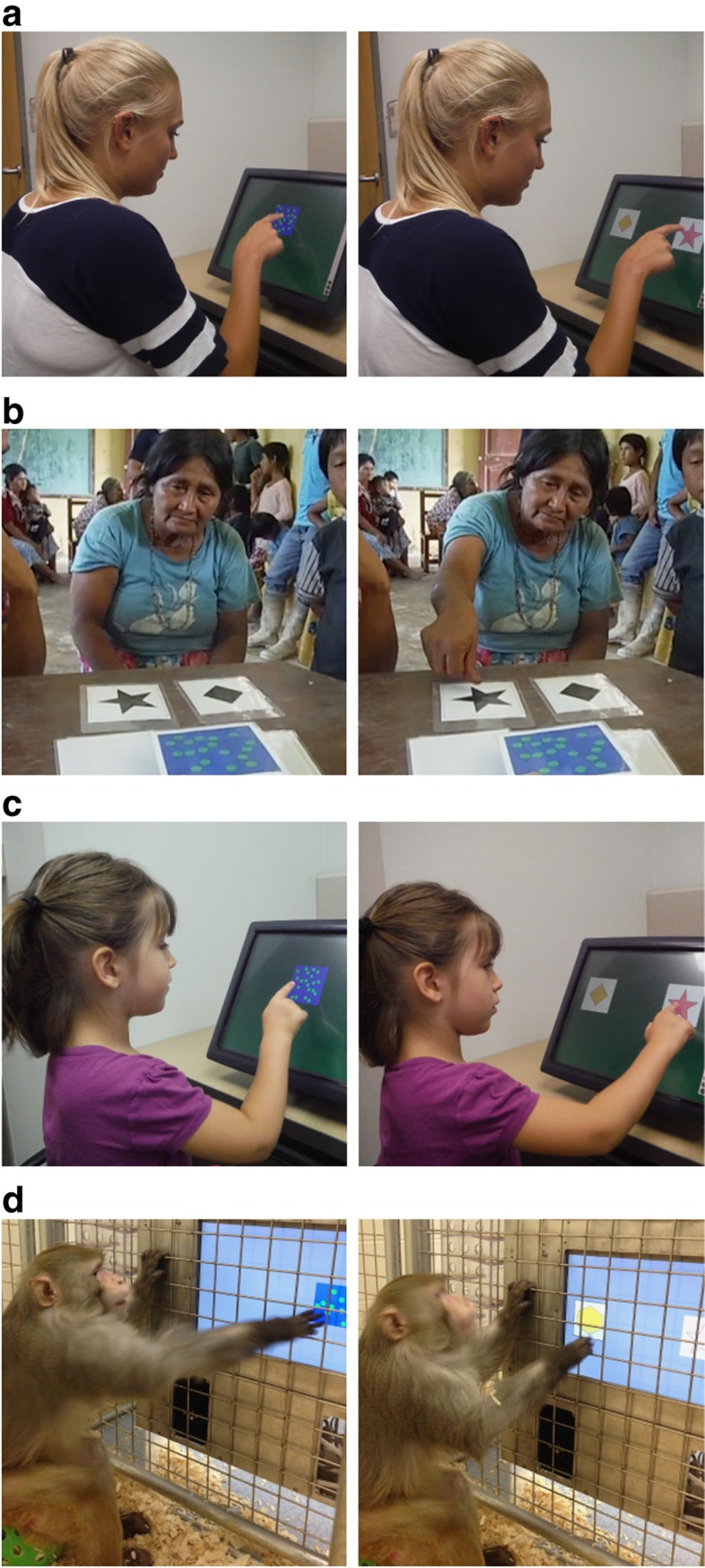
Subjects from each group tested in the categorization task. (**a**) US adults, (**b**) Tsimane' adults, (**c**) US children and (**d**) monkeys were trained to categorize dot arrays based on quantity. Subjects could use number, cumulative surface area or a combination of both dimensions as the basis of their category choices. We measured their spontaneous preferences for categorizing the quantities.

**Figure 2:**
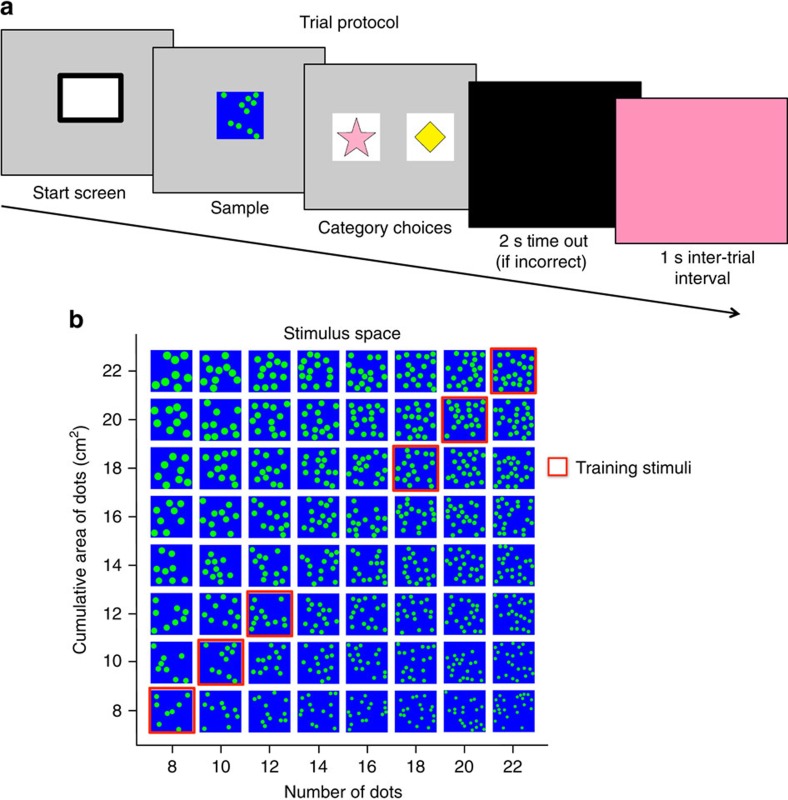
Trial protocol and stimulus space. (**a**) Example trial protocol. Subjects saw a neutral start screen, followed by presentation of a dot-array sample stimulus, followed by two choice icons for categorizing the dot-array as little (star) or a lot (diamond). During training, correct choices resulted in positive feedback, incorrect choices resulted in negative feedback. For probe stimuli, all choices were treated as correct responses. (**b**) Example stimulus space used in training (red outline) and testing (no outline).

**Table 1:** Relative contributions of number and cumulative area on classification.

**Group**	**Number weight (s.e.)**	**Cumulative area weight (s.e.)**	**Observations**
Monkeys	0.126***	0.027	380
	(0.027)	(0.143)	
4-5-year-olds	0.381***	0.095**	864
	(0.067)	(0.034)	
Tsimane' adults	0.681***	0.309***	714
	(0.081)	(0.054)	
US adults	0.805***	0.062	2,436
	(0.121)	(0.091)	

Note: *P<0.05; **P<0.01; ***P<0.001.

Beta weights (and s.e.) from mixed effects logistic regressions predicting ‘category choice' using ‘number' and ‘cumulative area' stimulus values as predictor variables and subject as a random effect. The coefficients have been centred but not standardized. The total number of probe observations is shown for each group (US adults: *n*=21, observations per subject=116; US 4-5-year-olds: *n*=27, observations per subject=32; Tsimane' adults: *n*=51, observations per subject=14; Monkeys: *n*=2, observations per subject=176 and 204).

## References

[b1] Gallistel C. R. The Organization of Learning MIT press (1990).

[b2] Gallistel C. R. & Gelman R. Preverbal and verbal counting and computation. Cognition 44, 43–74 (1992). 1511586 10.1016/0010-0277(92)90050-r

[b3] Gallistel C. R. & Gelman R. Non-verbal numerical cognition: from reals to integers. Trends Cogn. Sci. 4, 59–65 (2000). 10652523 10.1016/s1364-6613(99)01424-2

[b4] Cantlon J. F. & Brannon E. M. How much does number matter to a monkey (Macaca mulatta)? J. Exp. Psych: Anim. Behav. Proc 33, 32 (2007). 10.1037/0097-7403.33.1.32 17227193

[b5] Breukelaar J. W. & Dalrymple-Alford J. C. Timing ability and numerical competence in rats. J. Exp. Psych: Anim. Behav. Proc. 24, 84 (1998). 10.1037//0097-7403.24.1.84 9438968

[b6] Cantlon J. F. & Brannon E. M. Shared system for ordering small and large numbers in monkeys and humans. Psychol. Sci. 17, 401–406 (2006). 16683927 10.1111/j.1467-9280.2006.01719.x

[b7] Scarf D., Hayne H. & Colombo M. Pigeons on par with primates in numerical competence. Science 334, 1664–1664 (2011). 22194568 10.1126/science.1213357

[b8] Brannon E. M. The development of ordinal numerical knowledge in infancy. Cognition 83, 223–240 (2002). 11934402 10.1016/s0010-0277(02)00005-7

[b9] Izard V., Sann C., Spelke E. S. & Streri A. Newborn infants perceive abstract numbers. Proc. Natl Acad. Sci. USA 106, 10382–10385 (2009). 19520833 10.1073/pnas.0812142106 PMC2700913

[b10] Lipton J. S. & Spelke E. S. Origins of number sense large-number discrimination in human infants. Psychol. Sci. 14, 396–401 (2003). 12930467 10.1111/1467-9280.01453

[b11] Xu F. & Spelke E. S. Large number discrimination in 6-month-old infants. Cognition 74, B1–B11 (2000). 10594312 10.1016/s0010-0277(99)00066-9

[b12] Cordes S., Gelman R., Gallistel C. R. & Whalen J. Variability signatures distinguish verbal from nonverbal counting for both large and small numbers. Psychon. Bull. Rev. 8, 698–707 (2001). 11848588 10.3758/bf03196206

[b13] Frank M. C., Everett D. L., Fedorenko E. & Gibson E. Number as a cognitive technology: evidence from Pirahã language and cognition. Cognition 108, 819–824 (2008). 18547557 10.1016/j.cognition.2008.04.007

[b14] Pica P., Lemer C., Izard V. & Dehaene S. Exact and approximate arithmetic in an Amazonian indigene group. Science 306, 499–503 (2004). 15486303 10.1126/science.1102085

[b15] Strandburg-Peshkin A., Farine D. R., Couzin I. D. & Crofoot M. C. Shared decision-making drives collective movement in wild baboons. Science 348, 1358–1361 (2015). 26089514 10.1126/science.aaa5099 PMC4801504

[b16] Clearfield M. W. & Mix K. S. Number versus contour length in infants' discrimination of small visual sets. Psychol. Sci. 10, 408–411 (1999).

[b17] Feigenson L., Carey S. & Spelke E. Infants' discrimination of number versus. continuous extent. Cognit. Psychol. 44, 33–66 (2002). 11814309 10.1006/cogp.2001.0760

[b18] Pinel P., Piazza M., Le Bihan D. & Dehaene S. Distributed and overlapping cerebral representations of number, size, and luminance during comparative judgments. Neuron 41, 983–993 (2004). 15046729 10.1016/s0896-6273(04)00107-2

[b19] Cantrell L., Boyer T. W., Cordes S. & Smith L. B. Signal clarity: an account of the variability in infant quantity discrimination tasks. Dev. Sci. 18, (6): 877–893 (2015). 25601156 10.1111/desc.12283 PMC6448154

[b20] Cantrell L. & Smith L. B. Open questions and a proposal: a critical review of the evidence on infant numerical abilities. Cognition 128, 331–352 (2013). 23748213 10.1016/j.cognition.2013.04.008 PMC3708991

[b21] Mix K. S., Huttenlocher J. & Levine S. C. Multiple cues for quantification in infancy: is number one of them? Psychol. Bull. 128, 278 (2002). 11931520 10.1037/0033-2909.128.2.278

[b22] Holland P. C. Origins of behavior in Pavlovian conditioning. Psychol. Learn. Motiv. 18, 129–174 (1984).

[b23] Huanca T. Tsimane' Oral Tradition, Landscape, and Identity in Tropical Forest SEPHIS (2006).

[b24] Smith L. B. A model of perceptual classification in children and adults. Psychol. rev. 96, 125 (1989). 2928416 10.1037/0033-295x.96.1.125

[b25] Ashby F. G. & Maddox W. T. Integrating information from separable psychological dimensions. J. Exp. Psychol. Hum. Percept. Perform. 16, 598 (1990). 2144574 10.1037//0096-1523.16.3.598

[b26] Smith J. D. . Implicit and explicit categorization: a tale of four species. Neurosci. Biobehav. Rev. 36, 2355–2369 (2012). 22981878 10.1016/j.neubiorev.2012.09.003 PMC3777558

[b27] Gelman A. & Hill J. Data Analysis Using Regression and Multilevel/Hierarchical Models Cambridge University Press (2006).

[b28] Clogg C. C., Petkova E. & Haritou A. Statistical methods for comparing regression coefficients between models. Am. J. Sociol. 100, 1261–1293 (1995).

[b29] Feigenson L., Carey S. & Spelke E. Infants' discrimination of number versus. continuous extent. Cognit. Psychol. 44, 33–66 (2002). 11814309 10.1006/cogp.2001.0760

[b30] Mix K. S., Huttenlocher J. & Levine S. C. Multiple cues for quantification in infancy: is number one of them? Psychol. Bull. 128, 278 (2002). 11931520 10.1037/0033-2909.128.2.278

[b31] Gebuis T. & Reynvoet B. The interplay between nonsymbolic number and its continuous visual properties. J. Exp. Psychol.-Gen. 141, 642 (2012). 22082115 10.1037/a0026218

[b32] Burr D. & Ross J. A visual sense of number. Curr. Biol. 18, 425–428 (2008). 18342507 10.1016/j.cub.2008.02.052

[b33] Anobile G., Cicchini G. M. & Burr D. C. Number as a primary perceptual attribute: a review. Perception 45, 5–31 (2015). 26562858 10.1177/0301006615602599 PMC5040510

[b34] Dantzig T. Number: The Language of Science Penguin (2007).

[b35] Dehaene S., Dehaene-Lambertz G. & Cohen L. Abstract representations of numbers in the animal and human brain. TINS 21, 355–361 (1998). 9720604 10.1016/s0166-2236(98)01263-6

[b36] Boysen S. T., Berntson G. G., Hannan M. B. & Cacioppo J. T. Quantity-based interference and symbolic representations in chimpanzees (Pan troglodytes). J. Exp. Psych: Anim. Behav. Proc. 22, 76 (1996). 8568498

[b37] Cantlon J. F., Safford K. E. & Brannon E. M. Spontaneous analog number representations in 3-year-old children. Dev. Sci. 13, 289–297 (2010). 20136925 10.1111/j.1467-7687.2009.00887.x PMC2819667

[b38] Viswanathan P. & Nieder A. Neuronal correlates of a visual “sense of number” in primate parietal and prefrontal cortices. Proc. Natl Acad. Sci. USA 110, 11187–11192 (2013). 23776242 10.1073/pnas.1308141110 PMC3704030

[b39] Roitman J. D., Brannon E. M. & Platt M. L. Monotonic coding of numerosity in macaque lateral intraparietal area. PLoS Biol. 5, e208 (2007). 17676978 10.1371/journal.pbio.0050208 PMC1925133

[b40] Halberda J., Mazzocco M. M. & Feigenson L. Individual differences in non-verbal number acuity correlate with maths achievement. Nature 455, 665–668 (2008). 18776888 10.1038/nature07246

[b41] Park J. & Brannon E. M. Training the approximate number system improves math proficiency. Psychol. Sci. 24, 2013–2019 (2013). 23921769 10.1177/0956797613482944 PMC3797151

[b42] Marle K., Chu F. W., Li Y. & Geary D. C. Acuity of the approximate number system and preschoolers' quantitative development. Dev. Sci. 17, 492–505 (2014). 24498980 10.1111/desc.12143

[b43] Piazza M., Pica P., Izard V., Spelke E. S. & Dehaene S. Education enhances the acuity of the nonverbal approximate number system. Psychol. Sci. 24, 1037–1043 (2013). 23625879 10.1177/0956797612464057 PMC4648254

[b44] Cantrell L. & Smith L. B. Set size, individuation, and attention to shape. Cognition 126, 258–267 (2013). 23167969 10.1016/j.cognition.2012.10.007 PMC3749737

[b45] Peuskens H. . Attention to 3-D shape, 3-D motion, and texture in 3-D structure from motion displays. J. Cogn. Neurosci. 16, 665–682 (2004). 15165355 10.1162/089892904323057371

[b46] Brannon E. M., Lutz D. & Cordes S. The development of area discrimination and its implications for number representation in infancy. Dev. Sci. 9, F59–F64 (2006). 17059447 10.1111/j.1467-7687.2006.00530.x PMC1661837

[b47] Cordes S. & Brannon E. M. The difficulties of representing continuous extent in infancy: using number is just easier. Child Dev. 79, 476–489 (2008). 18366435 10.1111/j.1467-8624.2007.01137.x PMC2906149

[b48] Cordes S. & Brannon E. M. The relative salience of discrete and continuous quantity in young infants. Dev. Sci. 12, 453–463 (2009). 19371370 10.1111/j.1467-7687.2008.00781.x PMC2949063

[b49] Barth H., Kanwisher N. & Spelke E. The construction of large number representations in adults. Cognition 86, 201–221 (2003). 12485738 10.1016/s0010-0277(02)00178-6

[b50] Jordan K. E., Brannon E. M., Logothetis N. K. & Ghazanfar A. A. Monkeys match the number of voices they hear to the number of faces they see. Curr. Biol. 15, 1034–1038 (2005). 15936274 10.1016/j.cub.2005.04.056

[b51] Jordan K. E. & Brannon E. M. The multisensory representation of number in infancy. Proc. Natl Acad. Sci. USA 103, 3486–3489 (2006). 16492785 10.1073/pnas.0508107103 PMC1413880

[b52] Nieder A. Supramodal numerosity selectivity of neurons in primate prefrontal and posterior parietal cortices. Proc. Natl Acad. Sci. USA 109, 11860–11865 (2012). 22761312 10.1073/pnas.1204580109 PMC3406836

[b53] Ginsburg H. *The Test of early mathematics ability*. 3rd edn, (Austin, Texas, Pro-Ed (2003).

[b54] Baayen R. H., Davidson D. J. & Bates D. M. Mixed-effects modeling with crossed random effects for subjects and items. J. mem. lang. 59, 390–412 (2008).

[b55] Bates D., Maechler M., Bolker B. & Walker S. Fitting linear mixed-effects models using lme4. J. Stat. Softw. 67, 1–48 (2015).

[b56] Powell M. J. The BOBYQA algorithm for bound constrained optimization without derivatives. Cambridge NA Report, University of Cambridge, Cambridge (2009).

