# Supplementary material for: Universal and uniquely human factors in spontaneous number perception
Source: Nat Commun. 2017 Jan 16;8:13968. doi: 10.1038/ncomms13968 (PMC5241699; doi:10.1038/ncomms13968)
Supplement: Supplementary Information — Supplementary Figures, Supplementary Tables, Supplementary Notes, Supplementary Methods and Supplementary References [file ncomms13968-s1.pdf]

SUPPLEMENTARY FIGURES

Supplementary Figure 1

a

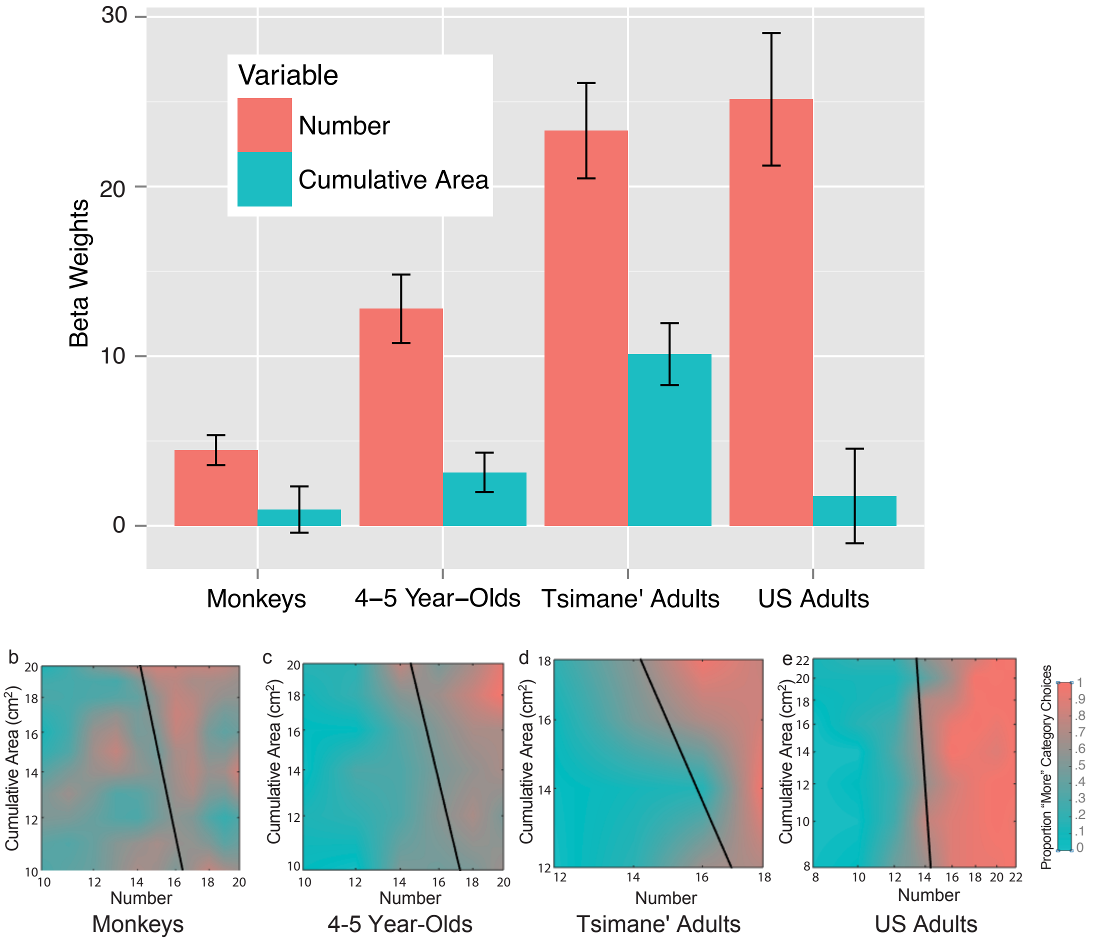

Supplementary Figure 1. Relative contributions of number and cumulative area on classification in log-log space. (**a**) Beta weights from a mixed effects logistic regression predicting category choice using log(number) and log(cumulative area) as predictor variables and including subject as a random effect (US adults: n = 21; Tsimane' Adults: n = 51, 4-5 year-old US children: n = 27 and Monkeys (n = 2). Error bars represent the standard error of the mean. (**b-e**) Heat maps and a linear classifier for monkeys (**b**), 4-5 year-old children (**c**), Tsimane' Adults (**d**), and US adults (**e**) in log-log space.

Supplementary Figure 2

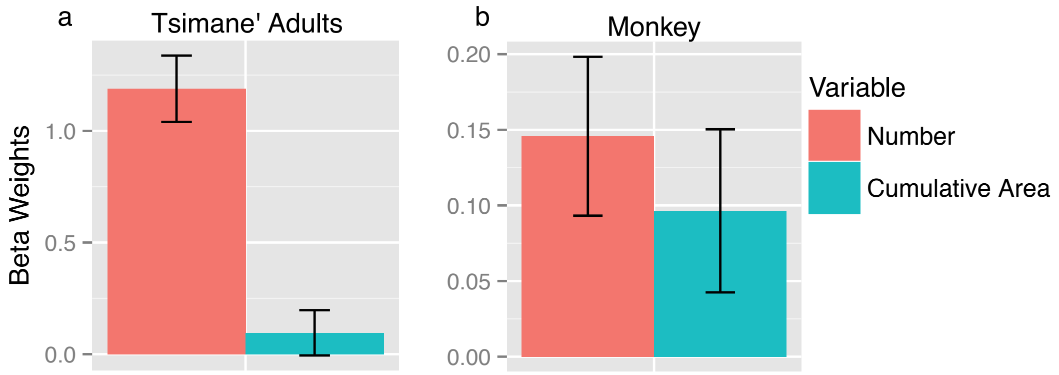

Supplementary Figure 2. Relative contributions of number and cumulative area in the density control experiment. Beta weights from the density control condition using a mixed effects logistic regression predicting category choice using number and cumulative area as predictor variables and including subject as a random effect for (a) Tsimane' adults (n = 31) and (b) Monkeys (n = 1). Error bars represent the standard error of the mean.

SUPPLEMENTARY TABLES

Supplementary Table 1

	Variable	Beta weight	SEM	Z	*p*-value
Humans vs. Monkeys	Number	0.496**	(0.160)	3.10	0.002
Humans vs. Monkeys	Area	0.128	(0.119)	1.07	0.283
Adults vs. Children	Number	0.362*	(0.158)	2.30	0.022
Adults vs. Children	Area	0.091	(1.112)	0.81	0.417
US Adults vs. Tsimane' Adults	Number	0.124	(0.145)	0.85	0.393
US Adults vs. Tsimane' Adults	Area	0.247*	(0.106)	-2.33	0.020
Note:	. p<.1; *p<.05; **p<.01; ***p<.001				

Supplementary Table 1. Group differences in number and cumulative area categorization biases. Group differences in number and cumulative area representations calculated by averaging coefficients from separate regressions and then comparing across groups (US adults: n = 21; Tsimane' Adults: n = 51, 4-5 year-old US children: n = 27 and Monkeys: n = 2). Standard error was approximated using the method of Clogg, Petkova, & Haritou (1995)[1]

Supplementary Table 2		
	Beta weight	SEM
Number	0.430***	(0.052)
Area	0.078**	(0.031)
Tema Score	0.492.	(0.257)
Age	0.448.	(0.262)
Number:Tema Score	0.099**	(0.049)
Area:Tema Score	-0.027	(0.030)
Number:Age	0.104**	(0.051)
Area:Age	-0.005	(0.031)
Constant	0.093	(0.259)
Note:	. p<.1; *p<.05; **p<.01; ***p<.001	

Supplementary Table 2. Effects of age and mathematics on number and area representations in US children. Beta weights (and SEM) from a mixed model logistic regression predicting category choice using number, area, and the interactions between Tema*Number, Tema*area, age*number and age*area as predictor variables and including subject as a random effect (Subjects = 32, observations = 1,023).

Supplementary Table 3		
	Beta weight	SEM
Number	0.683***	(0.079)
Area	0.309***	(0.054)
Education	-0.116	(0.173)
Number:Education	0.109	(0.073)
Area:Education	0.008	(0.052)
Constant	-0.211	(0.176)
Note:	. p<.1; *p<.05; **p<.01; ***p<.001	

Supplementary Table 3. Effect of education on number and area representations in Tsimane' adults. . Beta weights (and SEM) from a mixed model logistic regression predicting category choice using number, area, and the interactions between education*number and education*area and including subject as a random effect in Tsimane' adults (Subjects = 51, observations = 714).

Supplementary Table 4

Group	Number weight (SE)	Cumulative Area weight (SE)	Observations
Monkeys	0.146***	0.096.	149
	(0.053)	(0.054)	
Tsimane' Adults	1.189***	0.096.	434
	(0.149)	(0.101)	
Note:	. p<.1; *p<.05; **p<.01; ***p<.001		

Supplementary Table 4. Relative contributions of number and cumulative area on categorization in the density control. Beta weights (and SE) from the density control condition from mixed effects logistic regressions predicting 'category choice' using 'number' and 'cumulative area' stimulus values as predictor variables and subject as a random effect (Tsimane' adults n = 31; Monkeys n = 1). The coefficients have been centered but not standardized.

Supplementary Note 1

In the main text, we report that subjects have a bias to represent numerical information over cumulative surface area information. Here we test whether this bias to represent number could be explained by representing the density of the stimuli instead of the number of items. Previous research has shown that humans, and non-human animals can represent quantity even when density is equated across stimuli[2, 3]. However, recent work has shown that under some circumstances, human infants use density when making quantitative decisions[4]. It is unknown whether and to what degree humans and non-humans spontaneously use density when quantifying sets of objects. In Experiment 1 we showed that subjects did not use the density of the dots as a cue to the relative numerosity of the sets – subjects' category choices were not predicted by density independently of number. In Experiment 2 we tested whether subjects continue to show a number bias when density is equated across stimuli and thus completely unavailable as a category cue. If variation in density is critical for eliciting the number bias seen in Experiment 1, then we should not observe a number bias in Experiment 2. Alternatively, if subjects represent numerical value, then we should see a number bias even when density is neutralized.

Overall, subjects performed above chance on standard trials wherein number and area were correlated (one-sample Wilcoxon tests; Tsimane' adults: Mean = 90%, W = 1429, $p < .001$; Monkey by session: Mean = 78%, W = 561, $p < .001$). In order to determine the relative contributions of number and cumulative area to subjects' quantitative judgments when density was controlled, we conducted a mixed effects logistic regression for each group using number and area as predictor variables of category choice, and a random effects term of subject. As in Experiment 1, we found that number predicted category choice in both Tsimane' Adults and Monkeys (See figure S1, S2 & table S4). Also as in Experiment 1, area had a positive, but non-significant effect in both groups. We saw no decrease in number weights for either group in Experiment 1 versus Experiment 2 as would be expected if number is the primary basis for categorization. Instead we saw slight increases in both Tsimane' adults and Monkeys' number biases (Number weights: Tsimane' Adults: Exp. 1 = .68, Exp. 2 = 1.18; Monkey: Exp. 1 = .13, Exp. 2 = .14). These results show that variation in the density of the dots was not the cause of the number bias reported in Experiment 1. Instead subjects represent numerical information directly and spontaneously even when density is neutralized.

Supplementary Methods

Thirty-two Tsimane' adults (mean age = 33.5 years, standard deviation = 12.7 years, 7 male) and one monkey (from Exp. 1) were tested in the density control condition. One Tsimane' adult was excluded using the same criteria as Experiment 1 of chance performance on standard trials.

Procedure.

Training and testing procedures were identical to Experiment 1. The same values for number and cumulative surface area were tested, and were exactly the same as those from Experiment 1. Density was constant for all stimuli (both standard and probe) and could not be used as a cue for categorization during standard or probe trials. This was achieved by increasing the bounding box of the stimuli, such that the area of the bounding box for a 20-item dot array was twice as large as that of a 10-item dot array, and thus the number of items per cm^2 was constant. The size and color of the background remained constant across all stimuli.

SUPPLEMENTARY REFERENCES

1. Clogg, C. C., Petkova, E., & Haritou, A. Statistical methods for comparing regression coefficients between models. *Am. J. Sociol.*, 1261-1293 (1995).
2. Jordan, K. E. & Brannon, E. M. Weber's Law influences numerical representations in rhesus macaques (Macaca mulatta). *Anim. Cognition*, **9**(3), 159-172 (2006).
3. Cantlon, J. F., & Brannon, E. M. Shared system for ordering small and large numbers in monkeys and humans. *Psychol. Sci*. **17**(5), 401-406 (2006).
4. vanMarle, K. & Wynn, K. Tracking and quantifying objects and non-cohesive substances. *Dev. Sci*, **14**(3), 502-515 (2011).
